# Factors affecting screening for diabetic complications in the community: a multilevel analysis

**DOI:** 10.4178/epih.e2016017

**Published:** 2016-05-03

**Authors:** Jin A Han, Soo Jeong Kim, Gawon Kim, Eun Ji Kim, Soon Young Lee

**Affiliations:** 1 Department of Preventive Medicine and Public Health, Ajou University School of Medicine, Suwon, Korea; 2 Department of Health Administration, Dongseo University, Busan, Korea; 3 Gyeonggi Center for Hypertension and Diabetes, Suwon, Korea

**Keywords:** Diabetic complication, Multilevel analysis, Community health planning

## Abstract

**OBJECTIVES:**

The objective of the present study was to identify the factors that affect screening for diabetic complications by sex in the community.

**METHODS:**

This study used individual-level data from the 2013 Community Health Survey (CHS) for 20,806 (male, 9,958; female, 10,848) adults aged 30 years or older who were diagnosed with diabetes. Community-level data for 253 communities were derived from either CHS or national statistics. A chi-square test and multilevel logistic regression analysis was performed.

**RESULTS:**

There were significant differences in the rate of screening for diabetic complications according to individual-level and community-level variables. In the multilevel analysis, the community-level variance ratio of the null model was 7.4% and 9.2% for males and females, respectively. With regard to community-level variables, males were affected by the city type, number of physicians, and their living environment, while females were affected by number of physicians, natural and living environments, and public transportation.

**CONCLUSIONS:**

The factors that influenced individual willingness to undergo screening for diabetic complications differed slightly by sex; however, both males and females were more likely to undergo screening when they recognized their health status as poor or when they actively sought to manage their health conditions. Moreover, in terms of community-level variables, both males and females were affected by the number of physicians. It is essential to provide sufficient and ongoing opportunities for education on diabetes and its management through collaboration with local communities and primary care medical centers.

## INTRODUCTION

Uncontrolled blood glucose level in a diabetes patient is a critical risk factor of diabetic complications, and aggressive and consistent glycemic control decreases the occurrence and progression of microvascular and macrovascular complications of diabetes [1-4]. Therefore, glycemic control and regular screening for complications are essential for diabetes patients [5,6], but little research has been conducted to examine the rate of screening for diabetic complications. And the 2013 Korea Community Health Survey (CHS) showed that the rate of screening for diabetic complications 253 communities was very low and community variation of the screening rates was also high [7].

In order to reduce the burden of chronic diseases, the World Health Organization (WHO) has stressed in the Global Non-communicable Disease Action Plan that primary health care at the community-level should be coordinated with public health services [8].

To manage chronic diseases, Korea has been utilizing community-based services for the prevention and management of chronic diseases with cooperation from, and in association with, primary health care institutions in the community. A community intervention study conducted in 2013 by Gyeonggi Center for Hypertension and Diabetes has shown that in the patient group with counseling intervention on diabetes management, the rate of screening increased from 40.0% to 48.8% for retinopathy and from 33.6% to 41.6% for nephropathy compared to one year before [9]. The rate of screening for diabetic complications can increase even just with systematic counseling with patients in community. Accordingly, societal and economic burdens caused by diabetic complications will decrease if at the community-level the current rate of screening for diabetic complications and factors affecting the screening rate are identified, and based on the findings, appropriate systematic education and management services are provided.

Thus, in this study we aimed to examine the current rate of screening for diabetic complications among diabetes patients at the community level, and to investigate the effects of both individual-level and community-level characteristics on the screening rate by multilevel analysis.

## MATERIALS AND METHODS

### Data

For individual-level data, we used the original 2013 CHS data. Since 2008, CHS has been conducted every year in 253 regions. The survey population is non-institutionalized adults of age 19 or older residing in Korea. After a stratified sample of households is extracted with city/province, town/township/neighborhood, and housing type as stratifying variables, trained interviewers visit the sampled households and conduct the survey with adults of age 19 or older. The survey consists of 17 content areas including health status, health behaviors, and use of health care services [10].

Out of a total of 228,781 adults surveyed in 2013, 20,806 were included in the present study, who were 30 year of age or older, diagnosed with diabetes by a physician, and whose information was available in the dataset regarding whether they were screened for diabetic complications.

Community-level data of the 253 communities were constructed from the secondary data obtained from Statistic Korea and community annual statistical reports, as well as the standardized rates of communities that were computed by analyzing the original 2013 CHS data.

### Study variables

#### Dependent variable

##### Whether patients were screened for diabetic complications

The dependent variable in the study was whether a patient (aged 30 or more and diagnosed with diabetes by a physician) was screened for diabetic complication. Two survey questions were used to score the dependent variable: “In the last year, have you receivedan eye exam (eye fundus exam) to check whether a diabetic complication has occurred in the eyes?” and “In the last year, have you received a microurinetest (microalbuminuria test), other than a stick urine test, to check whether a diabetic complication has occurred in the kidneys (diabetic nephropathy)?” If a patient responded to at least one question with yes, he or she was scored as bring screened for diabetic complications.

#### Independent variables

##### Individual-level variables

For potential socioeconomic factors affecting patients’ screening behavior for diabetic complications, we included sex, age (30-39, 40-49, 50-59, 60-69, ≥70), monthly household income (<1 million Korean won [KRW], 1-2 million KRW, 2-3 million KRW, 3-4 million KRW, and >4 million KRW), education level (elementary school, middle school, higher than high school), and marital status (single, have spouse). For factors regarding health behaviors, the following were included: current smoking status (whether the patient was smoking daily or sometimes, if he or she had smoked a total of five packs of cigarettes throughout life), binge drinking behavior (whether the patient consumed a large quantity of alcohol in one sitting, i.e., seven glasses for male and five glasses for female, twice or more per week in the last year), a habit of regular exercise (whether in the last one week the patient performed high-impact exercise activities over 20 minutes per day for three days or more per week, or medium-impact exercise activities over 30 minutes per day for five days or more per week),and self rated health (whether the patient assessed his or her own health as “very good” or “good”). Additionally, with regard to disease management, the following factors were included: number of chronic diseases diagnosed by a medical doctor (including hypertension, dyslipidemia, stroke, myocardial infarction, and angina), number of blood glucose tests (the total number of tests in the last one year was divided by 12 to get the monthly number of blood glucose tests: <1, 1-4, 4-30, and >30), awareness of glycemic control (whether the patient thought his or her glucose level was appropriately controlled), awareness of glucose level (whether the patient was aware of his or her glucose level), diabetes treatment (whether the patient was currently treated with insulin, anti-diabetic drugs, or a non-pharmaceutical approach to manage glucose level), and education on diabetes management (whether the patient had received diabetes management education from a hospital/clinic, Korean traditional medical clinic, or a public health clinic).

##### Community-level variables

The community-level variables included in the study are as follows: city type (city and province), number of beds per 1,000 population, number of internists per 1,000 population, health and welfare budget allocation, financial autonomy, the extent of diabetes education or communication provided by health centers, community residents’ satisfaction with natural environments (such as air and water quality), community residents’ satisfaction with living environments (such as electricity, water and sewer services, trash collection, sports facilities), community residents’ satisfaction with health care facilities (such as public health centers, hospitals and private clinics, Korean traditional medical clinics, and pharmacies), community residents’ satisfaction with public transportation (bus, taxi, subway, train), and finally, standardized mortality of diabetes [7,11].

### Analytic procedure

All data analysis was performed using SPSS version 22.0 (IBM Corp., Armonk, NY, USA). Frequency analysis was conducted to examine the individual-level general characteristics of patients. To examine differences in the rates of screening for diabetic complications according to individual-level and community-level characteristics, chi-square tests were performed. For chi-square testing, community-level variables were categorized with the median as a cutpoint. Because means are sensitive to extreme values, the median is a more appropriate index to represent the group of data if the data has high variation [12], and local health statistics which shows community health levels also uses the median as a representative value [12,13].

In addition, in order to examine whether the screening behaviors for diabetic complications are affected by individual-level or community-level characteristics, multilevel logistic regression analysis was performed. To estimate community variation in the rate of screening for diabetic complications, multilevel analysis was conducted with a null model in which none of the individual-level and community-level variables were included; with model 1 in which individual-level variables were included; and finally with model 2 in which both individual-level and community-level variables were included. The intercept-only null model, i.e., a model without any explanatory variables, was a model to estimate the variance across the units of analysis, and we included it to ascertain whether or not community-level variables should be included to analyze the data. From the null model, variance components at different levels and intra-class correlations (ICCs) can be estimated. The formula for an ICC is as follows:

$$ICC=\frac{{\sigma^{2}}_{u0}}{{\sigma^{2}}_{u0}+{\sigma^{2}}_{e}}$$

${\sigma^{2}}_{u0}$: residual variance between communities

${\sigma^{2}}_{e}$: residual variance between individuals

Because individual-level residual variance cannot be estimated, ICC was estimated by replacing it with π^2^/3. The validity of the model was evaluated on the ratio of between-community variance to total variance, and we used ICC values of 5% to 25% as criteria [14,15]. Model 1 that includes individual-level variables and model 2 that includes both individual-level and community-level variables were full models, in which the influences of community-level variables as well as those of individual-level variables were tested. Akaike information criterion (AIC) were used as the informational criterion for multilevel model selection. The information criterion is based on −2 log likelihood, and a model with a lower value is judged to have a better fit than a model with a higher value, regardless of the rate of exponential decay [16]. Statistical significance was set at the level of p<0.05 in all cases.

## RESULTS

### General characteristics of study subjects

Of 20,806 subjects included in the present study, income and education levels were significantly lower in female compared to male. The rate of smoking was 17.6%, the rate of binge drinking was 19.4%, and the rate of regular exercise was 20.4%. Additionally, 70.3% had chronic diseases other than diabetes such as hypertension, myocardial infarction, angina, or stroke.

A high proportion of patients (87.3%) thought that their glucose level was under control, although only 6.5% measured their glucose level once a day. Male were more likely than female to know their glucose level (70.9% vs. 54.1%, respectively). Finally, 88.7% of study subjects were currently under treatment for diabetes; 32.0% had received diabetes management education, and 37.1% had been screened for diabetic retinopathy or diabetic nephropathy (Table 1).

### The rate of screening for diabetic complications according to individual-level and community-level characteristics

Considering individual-level characteristics, the rate of screening for diabetic complications was significantly higher in male than in female (38.5% vs. 35.8%, respectively). The screening rate was significantly lower in people in their 30’s and also those in their 70’s compared to other age groups. As income and education levels increased, the screening rate increased. Additionally, the screening rate was higher among people with a spouse compared to those without one. The screening rate was also higher among those who felt their health was poor (e.g., low self-rated health status, many chronic diseases other than diabetes, or awareness of uncontrolled glucose level) and among those who took actions to manage diabetes (e.g., a higher frequency of measuring blood glucose level, awareness of their glucose level, being treated for diabetes, and having received diabetes management education) (Table 2).

In regards to community-level characteristics, the screening rate was higher in cities, and also in communities with a higher level of medical resources, a higher level of financial support, a higher level of satisfaction with health care service, a higher level of satisfaction with public transportation, and finally, a lower rate of diabetes-related mortality (Table 3).

### Factors affecting diabetes patients’ screening behavior for diabetic complications

Table 4 show the results of multilevel logistic regression analysis in which factors affecting male and female diabetes patients’ screening behavior for diabetic complications were examined by considering both individual-level and community-level characteristics.

In the male’s null model, we estimated ICC was 0.074, which means that approximately 7.4% of the total variance was attributable to between-community variance. In model 1, in which individual-level variables were included, the following factors had significant effects on male diabetes patients’ screening behavior: income level, current smoking status, self-rated health status, number of chronic disease, frequency of measuring blood glucose level, awareness of glycemic control, awareness of their glucose level, diabetes treatment, and diabetes management education. In model 2, city type, number of internists in the community, and satisfaction with community living environments significantly influenced the rate of male diabetes patients’ screening behavior.

Regarding female diabetes patients’ screening behavior for diabetic complications, the random effect of the community was significant in all models, i.e., null model, model 1, and model 2, and between-community variance comprised over 5% of total variance in all cases. Individual-level factors affecting females’ screening behavior were the same as those identified in the male model in addition to age and education level. In the model in which community-level variables were also included, unlike the male model, city type was not significant, and instead, satisfaction with public transportation was significant.

## DISCUSSION

The present study aimed to examine the factors affecting individual patients’ screening behavior for diabetic complications and to provide empirical evidence with which to prioritize community health projects by considering not only individual-level characteristics but also community-level influences.

When the factors affecting individuals’ behaviors are present at a higher level such as the community, a general regression approach cannot accurately identify the nature of inter-variable associations and a multilevel regression approach must be used in order to investigate the effect of community characteristics [14,17]. In the present study, the ICC of the null model was estimated to be 7% in the male model and 9% in the female model, and the random effects of the community was significant, suggesting that the community-level factors should be included in the final models [14].

According to this study results, the rate of screening for diabetic complications was lower in patients in their 30’s and 70’s than in those in other age groups. A diabetes patient begins to show diabetic nephropathy symptoms approximately 15 years after the occurrence of diabetes, as the kidneys become impaired [5], and because of the lead time until symptoms manifest themselves, diabetes patients in their 30’s may not realize the need to screen for diabetic complications. Diabetes patients in their 70’s, in whom diabetes duration is likely to be longer and thus the risk of diabetic complications is higher, may mistake the symptoms for aging symptoms. Hence, it is urgent to provide them with adequate education on diabetes management and screening for diabetic complications.

Of community-level characteristics that influence individuals’ health, socioeconomic ones are very important. Communities with a higher socioeconomic level promptly spread health-related information and healthy behaviors, as well as control unhealthy behavior, and push for better health care services and facilities, through voluntary and collective behavior, all of which promote the health of community residents [4]. The present study found that although in the multilevel analysis the effect of community socioeconomic level on individuals’ screening behavior for diabetic complications did not reach statistical significance, in the univariate analysis the screening rate was higher in communities with an above-median socioeconomic level.

As shown in Table 4, although there were sex-specific factors affecting individuals’ screening behavior for diabetic complications, the screening behavior of diabetes patients of both sexes was affected by self-rated health status, aggressive or passive diabetes management, and the number of internists in community. These findings suggests the importance of a management system approach based on community and primary health care institutions that benchmarks Chronic Care Model (CCM) in the US and the Innovative Care for Chronic Conditions Framework of WHO [9].

Several studies have shown evidence for the effect of CCM. When CCM was applied to diabetes patients, a study conducted outside Korea showed significantly improved results of HbA1c, lipid profile, and diabetic urine, eye, and foot tests [18], and another study showed an improvement in patients’ self-management as well as an increase in educational programs provided to patients [19]. Yet another study has reported a significant improvement of diabetes-related outcomes including decreases in low-density lipoprotein, HbA1c, and blood pressure, suggesting that CCM was an effective delivery system in the management of chronic diseases in diabetes patients [20].

A study conducted in Korea has reported that as a consequence of a project to register and manage people at high risk for cardiovascular disease, the continuous treatment rate and utilization rate of medical clinics among outpatients increased [21]. Additionally, a study in which registered patients were followed up over five years reported that blood pressure significantly decreased and the proportion of patients whose glycemic control was managed increased [22]. In the present study, the extent to which chronic disease education and communication were provided by health care institutions did not affect the rate of screening for diabetic complications. Although those efforts were aimed at community residents overall rather than patients specifically, anyone could develop disease at any point in time, and therefore, at the community level, effective and appropriate input should be provided for residents to improve disease management ability.

In the present study, we investigated individual-level and community-level factors affecting male and female diabetes patients’ screening behavior for diabetic complications by using multilevel analysis, and identified sex-specific factors as well as common factors. Specifically, we found that in both male and female, the screening rate was higher among those who assessed their own health to be poor and who were aggressive in disease management. In addition, of community-level variables, the number of internists in the community affected both male and female patients’ screening behavior. These findings suggest that sufficient and consistent education on diabetes and how to manage it should be provided to patients through coordination between the community and primary health care institutions, and at the community-level, an adequate system aiming for community health promotion should be developed in coordination with primary health care institutions in order to enhance community residents’ disease management ability.

The present study examined only screening for retinopathy and nephropathy, and there may have been a bias because the survey was self-administered. Also, there were cases in which survey respondents were not aware of diabetes or of screening for diabetic complications, and accordingly the rates reported in the study could be underestimations. Moreover, the physical and socioeconomic environments of a community influence the quality of life and health of the members [23], and in the present study we used subjective assessments in lieu of objective measurements of the physical environments of a community, i.e., satisfaction with the natural and living environments and public transportation. Objective data on the physical environments such as climate and environmental pollution and foundational facilities like water, sewage, and roads are not yet available at the level of the city/county/district in Korea. Thus, it is suggested that follow-up research should construct objective indexes to measure the physical environments of a community.

## Figures and Tables

**Table 1.** Characteristics of the respondents according to sex

	ALL	Male	Female
Age^***^			
30-39	387 (1.9)	209 (2.1)	178 (1.6)
40-49	1,539 (7.4)	962 (9.7)	577 (5.3)
50-59	4,332 (20.8)	2,500 (25.1)	1,832 (16.9)
60-69	6,247 (30.0)	3,078 (30.9)	3,169 (29.2)
≥70	8,301 (39.9)	3,209 (32.2)	5,092 (46.9)
Monthly household income (10^6^ Korean won)^***^			
<1	6,899 (34.3)	2,480 (25.8)	4,419 (42.2)
1-2	4,575 (22.8)	2,323 (24.1)	2,252 (21.5)
2-3	2,963 (14.7)	1,562 (16.2)	1,401 (13.4)
3-4	2,123 (10.6)	1,191 (12.4)	932 (8.9)
>4	3,538 (17.6)	2,071 (21.5)	1,467 (14.0)
Education level^***^			
None	4,885 (23.5)	784 (7.9)	4,101 (37.8)
Elementary school	5,791 (27.9)	2,295 (23.1)	3,496 (32.3)
Middle school	3,231 (15.5)	1,853 (18.6)	1,378 (12.7)
Higher than high school	6,878 (33.1)	5,018 (50.4)	1,860 (17.2)
Married^***^	14,648 (70.3)	8,486 (85.2)	6,139 (56.6)
Current smoking^***^	3,662 (17.6)	3,266 (32.8)	396 (3.7)
Binge drinking^***^	1,952 (19.4)	1,844 (28.2)	108 (3.1)
Regular exercise^***^	4,232 (20.4)	2,482 (24.9)	1,752 (16.2)
Self rated health^***^	2,829 (13.6)	1,808 (18.2)	1,021 (9.4)
No. of chronic diseases^***^			
Only diabetes	6,161 (29.7)	3,305 (33.3)	2,848 (26.4)
Diabetes+1	9,234 (44.5)	4,209 (42.4)	5,010 (46.5)
Diabetes+2	4,357 (21.0)	1,937 (19.5)	2,413 (22.4)
D iabetes+3	991 (4.8)	477 (4.8)	512 (4.7)
No. of blood glucose tests (mo)^***^			
<1	4,314 (20.9)	2,074 (21.0)	2,240 (20.8)
1-4	11,771 (56.9)	5,277 (53.4)	6,494 (60.2)
4-30	3,245 (15.7)	1,807 (18.3)	1,438 (13.3)
>30	1,345 (6.5)	728 (7.4)	617 (5.7)
Awareness of glycemic control^***^	18,077 (87.3)	8,767 (88.5)	9,310 (86.3)
Awareness of glucose level^***^	12,903 (62.2)	7,051 (70.9)	5,852 (54.1)
Diabetes treatment^***^	18,463 (88.7)	8,757 (87.9)	9,706 (89.5)
Education on diabetes management^***^	6,652 (32.0)	3,313 (33.3)	3,339 (30.8)
Screening for diabetes complication^***^	7,715 (37.1)	3,833 (38.5)	3,882 (35.8)

Values are presented as number (%).

*** p<0.001.

**Table 2.** Screening of diabetic complication by individual-level characteristics

		All	Male	Female
Age	30-39	129 (33.3)	78 (37.3)	51 (28.7)
	40-49	624 (40.5)	367 (38.1)	257 (44.5)
	50-59	1,752 (40.4)	971 (38.8)	781 (42.6)
	60-69	2,542 (40.7)	1,292 (42.0)	1,250 (39.4)
	≥70	2,668 (34.6)	1,125 (35.1)	1,543 (30.3)
	p-value	< 0.001	< 0.001	<0.001
Monthly household income (10^6^ Korean won)	<1	2,193 (31.8)	837 (33.8)	1,356 (30.7)
	1-2	1,683 (22.6)	859 (37.0)	824 (36.6)
	2-3	1,194 (40.3)	641 (41.0)	553 (39.5)
	3-4	860 (40.5)	498 (41.8)	362 (38.8)
	>4	1,508 (42.6)	871 (42.1)	637 (43.4)
	p-value	< 0.001	< 0.001	<0.001
Education	None	1,350 (27.6)	233 (278)	1,127 (27.5)
	Elementary school	2,063 (35.6)	771 (33.6)	1,292 (37.0)
	Middle school	1,296 (40.1)	681 (36.8)	615 (44.6)
	Higher than high school	2,999 (43.6)	2,155 (42.9)	844 (45.4)
	p-value	< 0.001	< 0.001	<0.001
Married	Single	2,048 (33.1)	530 (36.0)	1,518 (32.2)
	Have spouse	5,667 (38.7)	3,303 (38.9)	2,364 (38.5)
	p-value	< 0.001	0.02	<0.001
Current smoking	Smoker	1,303 (35.6)	1,174 (35.9)	129 (32.6)
	Non-smoker	6,412 (37.4)	2,659 (39.7)	3,753 (35.9)
	p-value	0.02	< 0.001	< 1.00
Binge drinking	Doing	695 (35.6)	662 (35.9)	33 (30.6)
	Not doing	3,060 (37.6)	1,785 (38.0)	1,275 (37.2)
	p-value	0.05	0.06	0.09
Regular exercise	Doing	1,584 (37.4)	942 (38.0)	642 (36.6)
	Not doing	6,126 (37.0)	2,890 (38.7)	3,236 (35.6)
	p-value	0.31	0.27	0.21
Self rated health	Very good/good	876 (31.0)	589 (32.6)	287 (28.1)
	Moderate/bad/ very bad	6,839 (38.0)	3,244 (39.8)	3,595 (36.6)
	p-value	< 0.001	< 0.001	<0.001
No. of chronic diseases	Only diabetes	2,153 (35.0)	1,166 (35.3)	987 (34.7)
	Diabetes+1	3,081 (33.4)	1,512 (35.9)	1,569 (31.3)
	Diabetes+2	1,896 (43.6)	874 (45.1)	1,022 (42.4)
	Diabetes+3	546 (55.2)	270 (56.6)	276 (53.9)
	p-value	< 0.001	< 0.001	<0.001
No. of blood glucose tests (mo)	<1	1,326 (30.7)	630 (30.4)	696 (31.1)
	1-4	3,841 (32.6)	1,780 (33.7)	2,061 (31.7)
	4-30	1,601 (49.3)	916 (50.7)	685 (47.6)
	> 30	857 (63.7)	455 (62.5)	402 (65.2)
	p-value	< 0.001	< 0.001	<0.001
Awareness of glycemic control	Control	6,509 (36.0)	3,302 (37.7)	3,207 (34.4)
	Not controlled	1,185 (45.2)	522 (45.6)	663 (44.9)
	p-value	< 0.001	< 0.001	<0.001
Awareness of glucose level	Recognize	5,985 (46.4)	3,216 (45.6)	2,769 (47.3)
	Not recognized	1,708 (21.7)	608 (21.0)	1,100 (22.0)
	p-value	< 0.001	< 0.001	<0.001
Diabetes treatment	Yes	7,349 (39.8)	3,625 (41.4)	3,724 (38.4)
	No	366 (15.6)	208 (17.3)	158 (13.8))
	p-value	< 0.001	< 0.001	<0.001
Education on diabetes management	Yes	3,172 (47.7)	1651 (49.8)	1,521 (45.6)
	No	4,540 (32.1)	2,181 (32.8)	2,359 (31.4)
	p-value	< 0.001	< 0.001	<0.001

Values are presented as number (%).

**Table 3.** Screening of diabetic complications by community-level characteristics

		All	Male	Female
City type	Urban	5,146 (42.1)	2,597 (42.7)	2,549 (41.4)
	Rural	2,543 (30.0)	1,224 (31.9)	1,319 (28.4)
	p-value	< 0.001	<0.001	< 0.001
No. of beds per 1,000 population	High	3,985 (36.6)	1,950 (38.6)	2,035 (34.8)
	Low	3,704 (37.7)	1,871 (38.5)	1,833 (36.9)
	p-value	0.05	0.47	0.01
No. of internist per 1,000 population	High	3,943 (44.6)	1,996 (45.0)	1,947 (44.3)
	Low	3,746 (31.5)	1,825 (33.3)	1,921 (30.0)
	p-value	< 0.001	<0.001	< 0.001
Health and welfare budget allocation	High	3,662 (40.3)	1,822 (41.0)	1,840 (39.7)
	Low	4,027 (34.6)	1,999 (36.5)	2,028 (32.9)
	p-value	< 0.001	<0.001	< 0.001
Financial autonomy	High	3,822 (38.7)	1,916 (39.8)	1,906 (37.6)
	Low	3,867 (35.7)	1,905 (37.3)	1,962 (34.2)
	p-value	< 0.001	0.006	< 0.001
The extent of diabetes education or community provided by health centers	High	3,963 (38.1)	1,964 (39.0)	1,999 (37.2)
	Low	3,726 (36.1)	1,857 (38.0)	1,869 (34.4)
	p-value	< 0.001	0.164	0.002
Community residents’ satisfaction with natural environments	High	3,800 (33.2)	1,876 (35.0)	1,924 (31.5)
	Low	3,889 (42.0)	1,945 (42.7)	1,944 (41.3)
	p-value	< 0.001	<0.001	< 0.001
Community residents’ satisfaction with living environments	High	3,550 (36.1)	1,767 (37.4)	1,783 (34.9)
	Low	4,139 (38.0)	2,054 (39.6)	2,085 (36.6)
	p-value	0.002	0.01	0.03
Community residents’ satisfaction with health care facilities	High	3,876 (40.7)	1,927 (41.8)	1,949 (39.7)
	Low	3,813 (34.0)	1,894 (35.7)	1,919 (32.6)
	p-value	< 0.001	<0.001	< 0.001
Community residents’ satisfaction with public transportation	High	3,920 (42.5)	1,958 (43.2)	1,962 (41.7)
	Low	3,769 (32.8)	1,863 (34.6)	1,906 (31.2)
	p-value	< 0.001	<0.001	< 0.001
Standardized mortality of diabetes	High	3,647 (36.3)	1,791 (37.7)	1,856 (35.2)
	Low	4,042 (37.8)	2,030 (39.3)	2,012 (36.4)
	p-value	0.01	0.05	0.09

Values are presented as number (%).

**Table 4.** Factors affecting screening of diabetic complications based on multilevel analysis by sex

Variable		Male			Female			
		Null model	Model 1	Model 2	Null model	Model 1	Model 2	
Fixed effects								
Intercept (standard error)^***^		-0.46 (0.04)	-2.14 (0.25)	-2.38 (0.29)	-0.54 (0.04)	-3.05 (0.33)	-3.11 (0.39)	
Individual-level								
Age	30-39 (reference)							
	40-49		0.88 (0.60, 1.29)	0.89 (0.61, 1.30)		1.61 (0.97, 2.67)	0.63 (0.97, 2.73)	
	50-59		0.88 (0.62, 1.24)	0.89 (0.63, 1.26)		1.85 (1.13, 3.05)	1.81 (1.09, 3.00)	
	60-69		1.02 (0.71, 1.47)	1.04 (0.72, 1.49)		2.00 (1.18, 3.40)	1.91 (1.11, 3.27)	
	≥70		0.94 (0.65, 1.36)	0.94 (0.65, 1.37)		1.58 (0.94, 2.65)	1.50 (0.89, 2.53)	
Monthly household income (10^6^ Korean won)	< 1 (reference)							
	1-2		1.03 (0.86, 1.23)	1.01 (0.84, 1.21)		1.02 (0.81, 1.29)	0.98 (0.77, 1.24)	
	2-3		1.20 (0.97, 1.48)	1.16 (0.94, 1.42)		0.93 (0.72, 1.20)	0.87 (0.68, 1.12)	
	3-4		1.14 (0.91, 1.43)	1.09 (0.87, 1.36)		1.01 (0.75, 1.37)	0.95 (0.71, 1.28)	
	>4		1.22 (1.00, 1.48)	1.15 (0.94, 1.40)		1.43 (1.08, 1.88)	1.31 (1.00, 1.73)	
Education level	None (reference)							
	Elementary		0.97 (0.74, 1.28)	0.97 (0.73, 1.29)		1.15 (0.93, 1.43)	1.14 (0.91, 1.42)	
	Middle school		0.91 (0.68, 1.21)	0.88 (0.65, 1.18)		1.29 (0.97, 1.71)	1.24 (0.93, 1.66)	
	Higher than high school		1.14 (0.88, 1.48)	1.10 (0.84, 1.42)		1.47 (1.09, 1.98)	1.40 (1.03, 1.89)	
Married	Have spouse (reference)							
	Single		0.93 (0.79, 1.10)	0.92 (0.78, 1.09)		1.00 (0.83, 1.20)	0.98 (0.81, 1.18)	
Smoking	Non-smoker (reference)							
	Smoker		0.87 (0.77, 0.98)	0.86 (0.77, 0.98)		0.97 (0.70, 1.34)	0.94 (0.67, 1.30)	
Binge drinking	Not doing (reference)							
	Doing		0.95 (0.84, 1.07)	0.96 (0.85, 1.08)		0.64 (0.39, 1.05)	0.65 (0.39, 1.06)	
Regular exercise	Not doing (reference)							
	Doing		1.00 (0.89, 1.13)	1.02 (0.90, 1.15)		1.00 (0.81, 1.23)	1.025 (0.83, 1.27)	
Self rated health	Below moderate (reference)							
	Very good/good		0.77 (0.67, 0.89)	0.78 (0.68, 0.90)		0.64 (0.50, 0.81)	0.64 (0.50, 0.82)	
No. of chronic diseases	Only diabetes (reference)							
	2		1.08 (0.95, 1.23)	1.08 (0.94, 1.23)		1.03 (0.86, 1.25)	1.03 (0.85, 1.24)	
	3		1.39 (1.21, 1.61)	1.36 (1.17, 1.58)		1.45 (1.15, 1.83)	1.42 (1.12, 1.80)	
	4		2.12 (1.58, 2.85)	2.08 (1.55, 2.80)		1.72 (1.08, 2.73)	1.68 (1.05, 2.67)	
No. of blood glucose tests (mo)	< 1 (reference)							
	1-4		0.84 (0.72, 0.98)	0.86 (0.73, 1.00)		0.79 (0.64, 0.97)	0.81 (0.66, 1.00)	
	4-30		1.38 (1.15, 1.65)	1.41 (1.17, 1.70)		0.96 (0.73, 1.25)	0.98 (0.75, 1.28)	
	> 30		1.79 (1.41, 2.28)	1.79 (1.41, 2.28)		1.77 (1.17, 2.68)	1.74 (1.15, 2.63)	
Awareness of glycemic control	Not control (reference)							
	Control		0.71 (0.59, 0.85)	0.71 (0.59, 0.85)		0.61 (0.49, 0.76)	0.62 (0.50, 0.77)	
Awareness of glucose level	Not recognized (reference)							
	Recognized		2.42 (2.07, 2.82)	2.40 (2.06, 2.80)		2.41 (2.03, 2.86)	2.34 (1.96, 2.78)	
Diabetes treatment	No (reference)							
	Yes		2.83 (2.60, 3.46)	2.81 (2.29, 3.44)		5.09 (3.67, 7.07)	5.11 (3.67, 7.11)	
Education on diabetes management	No (reference)							
	Yes		1.79 (1.57, 2.03)	1.81 (1.59, 2.06)		1.51 (1.24, 1.83)	1.51 (1.24, 1.84)	
Community-level								
City type	Rural (reference)							
	Urban			1.31 (1.03, 1.67)			1.15 (0.84, 1.59)	
No. of beds per 1,000 population	Low than median (reference)							
	High than median			1.06 (0.91, 1.23)			0.96 (0.77, 1.20)	
No. of internist per 1,000 population	Low than median (reference)							
	High than median			1.26 (1.01, 1.57)			1.32 (1.01, 1.74)	
Health and welfare budget allocation	Low than median (reference)							
	High than median			1.02 (0.85, 1.23)			0.97 (0.77, 1.22)	
Financial autonomy	Low than median (reference)							
	High than median			1.05 (0.89, 1.23)			1.02 (0.82, 1.27)	
The extent of diabetes education or community provided by health centers	Low than median (reference)							
	High than median			0.99 (0.84, 1.16)			1.12 (0.91, 1.38)	
Community residents’ satisfaction with natural environments	Low than median (reference)							
	High than median			1.08 (0.85, 1.37)			1.02 (0.77, 1.36)	
Community residents’ satisfaction with living environments	Low than median (reference)							
	High than median			0.76 (0.64, 0.90)			0.80 (0.64, 1.00)	
Community residents’ satisfaction with health care facilities	Low than median (reference)							
	High than median			1.00 (0.81, 1.24)			0.72 (0.53, 0.98)	
Community residents’ satisfaction with public transportation	Low than median (reference)							
	High than median			1.11 (0.89, 1.39)			1.47 (1.07, 2.02)	
Standardized mortality of diabetes	Low than median (reference)							
	High than median			0.94 (0.80, 1.10)			0.90 (0.73, 1.12)	
Random effects								
Variance of community (standard error)^***^		0.26 (0.03)	0.21 (0.04)	0.17 (0.03)	0.33 (0.04)	0.31 (0.06)	0.29 (0.06)	
Akaike information criterion		43,127.16	27,935.65	27,936.87	47,518.24	15,255.56	15,265.97	
Intra-class correlation		0.07	0.06	0.05	0.09	0.09	0.08	

Values are presented as odds ratio (95% confidence interval).

*** p<0.001.
