# Supplementary material for: Factors affecting screening for diabetic complications in the community: a multilevel analysis
Source: Epidemiol Health. 2016 May 3;38:e2016017. doi: 10.4178/epih.e2016017 (PMC4914545; doi:10.4178/epih.e2016017)
Supplement: Supplementary file 1 [file epih-38-e2016017-supplementary.pdf]

연구논문(원저)

당뇨병 합병증검사 수진율에 영향을 미치는 요인: 다수준분석

한진아 [1), 김수정 [2), 김가원 [1), 김은지 [3), 이순영 [1)*]

1) 아주대학교 예방의학교실

2) 동서대학교 보건행정학과

3) 경기도 고혈압당뇨병 등록관리센터

* 16499 경기도 수원시 영통구 월드컵로 164 아주대학교 의과대학 예방의학교실

Tel: 031-219-5081 Fax: 031-219-5084 E-mail: solee5301@gmail.com

Factors affecting a screening for diabetic complication in community: A multi-level analysis

Jin A Han[1)], Soo Jeong Kim[2)], Ga Won Kim[1)], Eun Ji Kim[3)], Soon Young Lee[1)*]

1) Department of Preventive Medicine and Public Health , Ajou University school of Medicine

2) Department of Health Administration, Dongseo University

3) Gyeonggi center for hypertension and diabetes

* corresponding author: Department of Preventive Medicine and Public Health , Ajou University school of Medicine , 164 Worldcup-ro, Yeongtong-gu, Suwon-si, Gyeonggi-do, 16499, Korea

Tel: 031-219-5147 Fax: 031-219-5084 E-mail: solee5301@gmail.com

Running title: On the screening of diabetic complication in community

서론

국내의 당뇨병 유병률은 노령인구의 증가, 소아비만의 급증, 덜 움직이고 많이 먹는 생활습관이 만연하면서 급격히 증가하여 사회, 경제, 보건학적으로 심각한 문제가 되고 있다 [1]. 2013 년 국민건강영양조사결과에 따르면 공복혈당검사와 혈당조절약 복용을 기준으로 30 세 이상 당뇨병 유병률은 전체 11.9%로 이중 치료를 받고 있는 사람은 65.9%, 치료를 받는 사람 중에서도 혈당조절이 되고 있는 사람은 16.3%에 불과하였다 [2]. 우리 몸의 전신에 혈관이 분포하고 있으므로 지속적인 만성 고혈당은 여러 장기에 분포한 혈관에 합병증을 유발하기에 혈당관리가 매우 중요하다고 할 수 있지만[3-6] 이처럼 혈당관리가 제대로 되지 않아 각종 합병증이 초래되며 이로 인한 사회적 부담도 점차 커지고 있다 [1,7]. 이러한 당뇨병 합병증에는 대혈관 합병증으로 뇌, 심장, 말초혈관과 같은 대혈관 합병증이 있고, 눈, 콩팥, 신경에 손상을 일으키는 미세혈관 합병증이 있다 [8,9]. 특히, 우리나라 당뇨병 환자의 1/3 에서 당뇨병성 신증을 가지고 있으며, 1/10 에서 만성 콩팥병을 가지고 있었으며, 1/5 에서 당뇨병성 망막증이 있었다[10]. 이러한 당뇨병 합병증은 일단 발병되면 치료가 어려움으로 지속적인 혈당관리와 함께 정기적인 합병증 검사수진이 중요하다고 할 수 있다. 당뇨병으로 진단 받으면 최소한 6 개월에 한번은 안저검사[8]와 신장검사, 혈액검사[9] 를 받는 것을 권고하고 있다. 그러나 2013 년 지역사회건강조사 결과에 따르면 253 개 지역의 당뇨병 의사진단자의 안질환 합병증 검사수진율 중앙값은 30.9%, 신장질환 합병증 검사 수진율은 28.5%로 매우 낮은 수준이었고, 지역 간의 변이도 안질환 합병증 검사수진율의 경우 3.3%~60.0%, 신장질환 합병증 검사수진율은 1.3%~65.9%로 지역에 따른 큰 차이가 있었다 [11]. 이처럼 개인의 건강과 관련된 행위는 개인의 특성 뿐 아니라 개인을 둘러싼 환경에 의해서도 영향을 받게 되는데 [12,13], 개인의 행태에 영향을 미치는 요인이 개인 수준이 아닌 지역과 같은 상위 수준에 존재할 경우 일반적인 회귀분석 방법으로는 정확한 연관성을 파악할 수 없으므로 지역 특성의 영향력을 분석하고자 할 경우에 다수준 분석을 사용하는 것이 적절하다고 할 수 있다[13-15].

한편, Global NCD(non-communicable disease) Action Plan 에 의하면 만성질환의 질병부담을 줄이기 위해 지역사회 내에서의 1 차 의료와 공공의료의 협업을 강조하였다[16]. 국내에서는 만성질환관리를 위해 지역사회에서 1 차 의료기관과의 협력과 연계하에 지역사회 기반의

만성질환 예방 및 관리서비스를 적용 실시하고 있는데, 2013 년 경기도 고혈압·당뇨병 등록관리센터에서 시행한 지역사회 개입연구 결과를 보면 당뇨병 상담개입군에 있어서 안질환 검사수진율 40.0%, 신장질환 검사율 33.6%이었던 것이 1 년 후 48.8%, 41.6%로 증가하였다[17]. 환자에 있어서 체계적인 상담교육만 잘 이루어져도 합병증 검사 수진율이 증가하므로 지역사회에서 당뇨병 환자들의 합병증 검사 수진율을 파악하고 이에 영향을 미치는 요인을 파악하여 이에 따른 적절하고 체계적인 교육 및 관리를 한다면 당뇨병 합병증으로 인한 사회적, 경제적 부담을 줄일 수 있을 것이다.

따라서 본 연구는 지역사회의 당뇨병 환자들의 합병증 검사 수진율을 파악하고 개인의 특성과 지역사회의 특성이 합병증 검사 수진여부에 어떻게 영향을 미치는지 살펴보고자 하였다.

연구방법

연구자료

개인수준의 데이터는 2013 년 지역사회건강조사 원데이터를 사용하였다. 2008 년 이후로 매년 전국 253 개 시군구 지역에서 실시하고 있는 지역사회건강조사는 각 지역의 19 세 이상 성인을 모집단으로 하고 시/군, 동/읍면, 주택유형을 층화 변수로 하여 표본가구를 추출한 후 숙련된 조사원이 표본가구를 방문하여 만 19 세 이상 성인을 대상으로 건강수준, 건강행위, 의료이용 등 총 17 개 영역에 대해 면접조사를 수행한다 [18]. 2013 년 조사응답자 228,781 명 중 30 세 이상 당뇨병 의사진단 경험자이면서 당뇨병 합병증 검사수진 여부에 대한 정보가 있는 20,806 명이 개인단위 분석에 사용되었다. 지역단위의 데이터는 통계청과 각 지역의 통계연보를 통해 얻어진 2 차 자료와 2013 년 지역사회 건강조사 원데이터 분석하여 얻어진 각 지역의 표준화율을 사용하여 253 개 지역별 데이터를 구축하였다.

연구변수

종속변수: 당뇨병 합병증검사수진여부

종속변수로 사용된 당뇨병 합병증 검사수진여부 변수는 30 세 이상 당뇨병 의사진단 경험자 중 “최근 1 년 동안 당뇨병으로 인한 눈의 합병증이 발생했는지 확인하기 위해

눈검사(안저검사)를 받은 적이 있습니까?", "최근 1 년 동안 당뇨병으로 인한 신장(콩팥) 합병증이 발생했는지 확인하기 위해 스틱검사를 제외한 정밀소변검사(미세단백뇨 검사)를 한 적이 있습니까"라는 설문 문항 중 하나라도 그렇다고 응답한 사람을 당뇨병 합병증 검사수진을 한 경우로 정의하였다.

독립변수

개인수준변수

합병증 검사 수진행위에 영향을 미칠 수 있는 개인의 사회경제학적 요인으로 성별, 연령(30-39 세/ 40-49 세/ 50-59 세/ 60-69 세/ 70 세 이상), 학력(초졸/ 중졸/ 고졸/ 대학이상), 가구소득수준(100 만원 미만/ 100-200 만원/ 200-300 만원/ 300-400 만원/ 400 만원 이상), 배우자여부(없음-미혼·이혼·별거·사별/ 있음)변수를 사용하였고, 건강행태 요인으로는 현재흡연여부(평생 5 갑 이상 피운 사람 중에 현재 매일 피우거나 가끔 피우는 여부), 고위험음주여부(최근 1 년 동안 한 번의 술자리에서 남자는 7 잔, 여자는 5 잔 이상을 주 2 회 이상 마시는 여부), 중등도 이상 운동실천여부(최근 1 주일 동안 격렬한 신체활동을 1 일 20 분 이상, 주 3 일 이상 실천하거나 중등도 신체활동을 1 일 30 분 이상 주 5 일 이상 실천하는 여부), 주관적 양호한 건강상태 여부(주관적 건강수준을 '매우 좋음' 또는 '좋음'이라고 응답하는 여부) 변수를 사용하였다. 또한 질병관리 요인으로는 의사에게 진단받은 만성질환 개수(의사에게 고혈압 또는 고지혈증을 포함한 이상지질혈증 또는 뇌졸중 또는 심근경색증 또는 협심증 중 진단 받은 질환 개수), 혈당 측정횟수(지난 1 년 동안 혈당 측정 횟수를 12 로 나누어 월별 혈당 측정횟수를 산출함: 1 회 미만/1-4 회/ 4-30 회/30 회 이상), 자가혈당조절인지 여부(본인의 혈당이 잘 조절되고 있는지에 대한 여부) , 혈당수치인지 여부(본인의 혈당수치를 알고 있는지에 대한 여부), 당뇨병 치료여부(현재 혈당관리를 위해 인슐린주사 또는 당뇨병 약 또는 비약물요법을 통해 치료를 받고 있는지에 대한 여부), 관리교육이수 여부(당뇨병 관리하는 방법에 대해 병의원 또는 한방병의원 또는 보건소에서 교육을 받은 적이 있는지에 대한 여부) 변수를 사용하였다.

지역수준변수

지역수준변수로는 도시유형(시/군), 인구 천명당 병상 수(국가통계포털: e-지방통계, 2013), 인구 천명당 내과 전문의 수(국가통계포털: 건강보험통계, 2013), 보건복지 예산

비중(지방자치단체 통계연보, 2012), 재정자주도 변수(국가통계포털: e-지방지표, 2013) 를 사용하였다. 또한 보건기관 당뇨병 교육 및 홍보 경험률(지역사회건강조사, 2013), 지역주민의 우리동네 자연환경(공기질, 수질 등) 만족도(지역사회건강조사, 2013), 생활환경(전기, 상하수도, 쓰레기 수거, 스포츠시설 등) 만족도(지역사회건강조사, 2013), 의료서비스 여건(보건소, 병의원, 한방병의원, 약국 등) 만족도(지역사회건강조사, 2013), 대중교통 여건(버스, 택시, 지하철, 기차 등) 만족도(지역사회건강조사, 2013), 당뇨병 질환 사망률 변수(국가통계포털: 사망원인통계, 2013)를 사용하였다.

분석방법

SPSS 22.0 버전을 사용하여 분석을 수행하였다. 본 연구에 포함된 연구대상자에 있어서 개인수준의 일반적인 특성을 살펴보기 위하여 빈도분석을 실시하였고 지역의 일반적인 특성을 살펴보기 위하여 지역단위 변수의 최저값, 최고값, 평균, 중앙값, 극단치의 비(Extremal Quotient, EQ), 변이계수를 산출함으로 지역 간 분포를 파악하였다. EQ 는 최소값과 최대값의 비이기 때문에 자료 전체의 성격보다는 극단치를 파악하는데 유용하고 변이계수는 표준편차와 평균의 비로 극단치에 의한 영향은 EQ 보다 덜 민감하며 전체 자료의 변이에 대한 정보를 제시할 수 있다 [19]. 개인적 특성 및 지역적 특성에 따른 당뇨병 합병증 검사 수진율의 차이를 살펴보기 위하여 카이제곱분석을 수행하였고 이때 지역단위 변수는 중위수를 기준으로 범주화하였다. 평균값의 경우 극단치에 민감한 영향을 받기 때문에 변이수준이 큰 자료에서는 집단을 대표하는 값으로 중위값이 더 적합하다고 판단하고 있으며[20], 지역의 건강수준을 나타내는 지역건강통계에서도 중위수를 사용하여 대표값을 제시하고 있다[20,21]. 또한 당뇨병 합병증 검사를 받는 것이 개인의 특성에 따른 것인지 지역사회 특성에 의해 영향을 받는지 확인하기 위해 다수준 로지스틱 회귀분석을 수행하였다. 당뇨병 합병증 검사 수진율의 지역간 변이를 추정하기 위해 개인수준과 지역수준 변수 모두를 포함하지 않은 기초모델(Null model), 개인수준 변수를 포함한 Model 1, 개인수준과 지역수준 변수를 모두 포함한 Model 2 를 구축하여 다수준 분석을 수행하였다. 설명변수가 없는 모형, 즉 절편만 있는 Null model 은 분석단위별 분산을 추정하는 모형이며, 본 연구에서는 지역 수준을 반영하여 분석하는 것이 필요한지를 확인하였다. Null model 에서는 각

수준별 분산성분과 집단 내 상관계수(intra-class correlation, ICC)를 알 수 있으며, ICC 를 구하는 공식은 다음과 같다.

$$\mathrm{ICC} = \frac{\sigma^2_{u0}}{\sigma^2_{u0} + \sigma^2_{e}}$$

σ^2_{u0} : 지역간 잔차 분산, σ^2_{e} : 개인간 잔차분산

개인 수준의 잔차분산이 산출되지 않아 $\pi^2/3$ 을 대신하여 ICC 값을 산출하였다. 총 분산에 대한 집단 간 분산 비율 판정을 통해 모델 설정의 타당성을 살펴보았고 이때 단서가 되는 ICC 값은 5~25%를 기준으로 하였다 [14,22]. 개인수준의 변수를 포함한 Model 1 과 개인수준과 지역수준의 변수를 모두 포함한 Model 2 는 Full model 로 개인수준 변수의 영향력은 물론 지역수준 변수들의 영향력을 제시하는 모형이다. 또한 다수준분석에서 사용하는 정보기준에는 AIC(Akaike Information Criterion) 를 사용하였다. 정보기준은 -2 로그우도에 근거하며 지수감소량과는 관계없이 낮은 값을 가지는 모형이 높은 값을 가지는 모형에 비해 개선되었다고 판단할 수 있다[23]. 모든 통계적 유의성의 판단은 유의수준 0.05 를 기준으로 하였다.

연구결과

1. 연구 대상자의 일반적인 특성

2013 년 지역사회 건강조사 대상자 중 30 세 이상 당뇨병 평생 의사진단경험자는 총 203,954 명 중 20,848 명으로 10.2%이었다. 이 중 본 연구의 대상자 20,806 명 중 남성은 47.9%, 여성은 52.1%이었고, 연령대가 높아질수록 당뇨병 의사진단자 비율이 높았다. 소득수준은 1,000,000 원 이하가 34.3%로 가장 많았고, 교육수준은 대졸, 초졸, 무학이 33.1%, 27.9%, 23.5% 순으로 많았으며, 여성의 경우 남성에 비해 소득수준과 교육수준이 유의하게 낮았다. 유배우자인 경우가 70.3%이었다. 흡연율은 17.6%, 고위험음주율은 19.4%, 중등도 이상 신체활동 실천율은 20.4%, 주관적 양호한 건강수준 인지율은 13.6%이었다. 또한 응답자 중 70%가 당뇨병 이외에도 고혈압, 심근경색증, 협심증, 뇌졸중 과 같은 만성질환을 함께 앓고 있었다. 1 일 1 회 혈당 측정을 하는 응답자는 6.5%에 불과하였으나 본인의 혈당이 조절되고 있다고 생각하는 경우가 87.3%로 높은 편이었고, 본인의 혈당수치를 알고 있는 경우가 남성 70.9%, 여성 54.1%로 남성에 있어서 높았다. 또한 당뇨병 의사진단자 중에서 88.7%가 현재 치료를 하고 있으며, 32.0%가 당뇨병

관리교육을 받은 경험이 있고, 37.1%가 안질환이나 신장질환 합병증 검사를 받은 적이 있다고 응답하였다<Table 1>.

2. 지역단위 변수의 지역간 분포

253 개 지역 중 시지역이 169 개(66.8%), 군지역이 84 개(33.2%)이었다. 지역변수의 지역간 분포는 <Figure 1>과 같이 그 변이가 큰 것을 볼 수 있다. 특히, 인구 1000 명당 내과 전문의 수는 지역 간 최소값과 최대값의 비를 나타내는 EQ 가 467.8 로 변수들 중 극단치의 비가 가장 큰 변수이었다. 당뇨병 교육홍보 경험률과 지역의 보건예산의 비중, 당뇨병 연령표준화 사망률 역시 가장 낮은 지역과 높은 지역의 차이가 약 10 배 이상으로 지역 간 편차가 큰 변수이었다. 또한 표준편차를 평균으로 나눈 값으로 나타낸 변이계수(coefficient of variation)는 인구 십만명당 내과 전문의 수가 1.2 로 다른 변수들에 비해 상대적으로 산포도가 큰 변수이었다.

3. 개인적/지역적 특성에 따른 당뇨병 합병증 검사 수진율

개인적 특성에 따른 당뇨병 합병증 검사 수진율은 남자 38.5%, 여자 35.8%로 남자에서 유의하게 높았고 연령대별로는 30 대와 70 세 이상에서 다른 연령에 비해 유의하게 낮았다. 소득수준과 교육수준이 높을수록, 배우자가 있는 경우 합병증 검사 수진율도 유의하게 높았다. 본인의 현재 건강상태가 좋지 않다고 느끼는 경우(예: 건강수준이 나쁘다고 인지할 때, 당뇨병 이외에 진단받은 만성질환 개수가 많을 때, 본인의 혈당이 조절되고 있지 않다고 인지할 때)와 당뇨병 관리를 위한 행동을 취하는 경우(예: 혈당을 더 많이 측정할수록, 본인 혈당수치 인지할 때, 치료를 받거나 관리교육을 받은 경험이 있음)에 합병증 검사수진율이 높았다<Table 2>. 지역적 특성에 따른 합병증 검사 수진율은 도시지역에 거주할 때, 지역의 의료자원이나 재정적 지원, 거주지역의 의료서비스나 교통시설에 만족도가 높은 지역에 거주할 때 높았으며, 당뇨병으로 인한 사망률이 낮은 지역에서 합병증 검사수진율이 높았다<Table 3>.

4. 당뇨병 합병증 검사 수진행위에 영향을 미치는 요인

<Table 4>와 <Table 5>는 성별에 따라 당뇨병 합병증 검사수진행위에 영향을 미치는 요인을 개인수준과 지역수준의 요인을 함께 고려하여 분석한 다수준 로지스틱 회귀분석의 결과이다. 30 세 이상 남성의 당뇨병 의사진단자의 합병증 검사수진여부에 지역간 변이가 있는지 파악한 Null model 에 따르면 개인수준과 지역수준의 변수를 고려하지 않아도 남성의 당뇨병 합병증 검사수진행위의 지역수준 분산은 0.264 이고, 지역 변량효과에 유의한 차이가 있었다(p<0.001). ICC 는 0.074 로 지역수준의 분산이 총 분산에서 차지하는 비율이 약 7.4%이었다. 개인수준의 변수를 투입한 Model 1 의 결과 남성의 당뇨병 합병증 검사 수진행위에는 소득수준, 흡연여부, 주관적 건강수준 인지여부, 만성질환 진단 갯수, 혈당측정횟수, 혈당조절 인지 및 본인 혈당수치 인지여부, 당뇨병 치료와 관리교육 이수 여부가 영향을 주었다. 지역수준 분산은 0.210 이고, 지역의 변량효과에 유의한 차이가 있었으며(p<0.001), ICC 는 0.060 이었다. Model 1 에 지역변수를 투입한 Model 2 의 결과 남성의 당뇨병 합병증 검사행위에 영향을 미치는 지역변수는 도시유형, 내과전문의 수, 생활환경 만족도이었다. 지역수준 분산은 0.170 이고, 지역의 변량효과에 유의한 차이가 있었으며(p<0.001), ICC 는 0.049 이었다. 개인수준 변수를 투입한 모델에서 지역수준이 설명된 분산량은 20.5%(=(0.264-0.210)/0.264)이었고, 개인수준과 지역수준의 변수를 모두 투입한 모델에서 지역수준이 설명된 분산량은 35.6%(=(0.264-0.170)/0.264)로 지역수준의 변수들을 함께 고려했을 때 당뇨병 합병증 검사수진에 대한 분산을 더 잘 설명해준다.

여성의 당뇨병 합병증 검사수진 행위 또한 Null model 과 Model1, Model2 모두 지역 변량효과에 유의한 차이가 있었고, 지역수준의 분산이 총 분산에서 차지하는 비율이 모두 5%이상이었다. 개인수준에서 당뇨병 합병증 검사 수진행위에 영향을 미치는 요인은 남성의 경우와 동일하였고 연령과 학력수준이 추가적으로 영향을 미치는 것을 확인할 수 있었다. 지역수준의 변수를 함께 투입한 모델에서는 남성과는 다르게 도시유형이 유의한 영향을 미치지 않았고 교통시설에 대한 만족도가 유의한 영향을 미쳤다. 개인수준 변수를 투입한 모델에서 지역수준이 설명된 분산량은 6.6%(=(0.332-0.310)/0.332)이었고, 개인수준과 지역수준의 변수를 모두 투입한 모델에서 지역수준이 설명된 분산량은 13.6%(=(0.332-0.287)/0.332)로 지역수준의 변수들을 함께 고려했을 때 당뇨병 합병증 검사수진에 대한 분산을 더 잘 설명해준다.

고찰

본 연구는 지역사회건강조사 자료를 활용하여 30 세 이상 당뇨병 의사진단경험자의 합병증 검사 수진행위에 영향을 미치는 요인을 성별에 따라 다수준 로지스틱 회귀분석을 통해 살펴보았다. 당뇨병 환자의 혈당관리여부는 합병증 발생의 매우 큰 위험요인이며 적극적이고 지속적인 혈당조절은 당뇨병 합병증인 미세혈관 합병증과 대혈관 합병증의 발생 및 진행을 낮춘다[3-6]. 혈당관리와 함께 정기적인 합병증 검사가 필수적[8,9]이나 합병증 검사 수진에 대한 연구가 매우 부족한 실정이다. 따라서 본 연구는 개인의 합병증검사 수진 행위에 영향을 미치는 요인에 대해 개인적 특성 뿐 아니라 지역적 특성까지 고려함으로 지역의 보건사업 우선순위 설정의 근거를 제시하고자 하였다.

개인의 행태에 영향을 미치는 요인이 지역과 같은 상위 수준에 존재할 경우 일반적인 회귀분석 방법으로는 정확한 연관성을 파악할 수 없으므로 지역 특성의 영향력을 살펴보고자 할 경우에는 다수준 분석을 수행하는 것이 적절하다[13-15]. 본 연구에서는 당뇨병 합병증 검사수진율에 대한 다수준 분석 결과 기초모델의 ICC 값이 남성 7.4%, 여성 9.2%이었고 지역변량의 효과가 유의하게 나타나 지역수준까지 모형에 포함할 근거가 되었다 [14].

당뇨병 의사진단 경험자의 88.7%가 현재 당뇨병을 치료하고 있지만 이들 중 32.0%만이 당뇨병 관리교육을 받았고 37.1%만이 신장질환 및 안질환 합병증 검사를 받았다. 특히, 30 대와 70 세 이상에서 합병증 검사 수진율이 각각 33.3%, 34.6%로 다른 연령대에 비해 낮았다. 당뇨병성 신증은 당뇨병 발병 후 15 년 정도가 되면 콩팥에 손상이 생기면서 증상이 나타나게 되는데 [8], 30 대에서는 이러한 합병증 증상발현까지의 lead time 으로 합병증 검사 수진에 대한 필요성을 느끼지 못할 것이다. 당뇨병 교육유무에 따른 합병증 검사 수진율에는 통계적으로 유의한 차이가 있었는데 특히 70 세 이상 의사진단자 중 당뇨병 관리교육을 받은 경우가 28.6%에 불과하여 다른 연령대에 비해 매우 낮았다. 2006 년 국내 3 차 기관급에서 당뇨병 조절 상태와 당뇨병 합병증 유병률에 대해 조사한 결과 미세알부민뇨를 보이는 환자는 30.4%, 망막병증은 38.3%의 유병률을 보이고 이러한 합병증은 당뇨조절 상태가 좋지 않을수록, 당뇨병 유병기간이 길수록 증가하였다 [7]. 이처럼 당뇨병 유병기간이 긴 확률이 높은 70 세 이상 당뇨병 환자들은

당뇨병 합병증 위험이 높음에도 불구하고 이러한 합병증 증상을 노화로 인한 증상으로 받아들일 수가 있어 이에 대한 적절한 질병관리 및 합병증 검사에 대한 교육이 시급하다고 할 수 있다.

개인의 건강에 영향을 미칠 수 있는 지역적 특성 가운데 지역의 사회경제적인 특성은 매우 중요한 특성이다. 지역의 사회경제적 수준이 높은 지역은 자발적이고 집단적인 행동을 통해 건강관련 정보와 건강에 좋은 행동양식을 빠르게 확산시키며, 건강에 악영향을 줄 수 있는 행동을 통제하고 보건의료 서비스와 관련 시설을 더 잘 갖추도록 함으로 지역에 거주하는 개개인들의 건강을 증진시킨다[6]. 본 연구의 다수준 분석의 결과에 따르면 지역의 사회경제적 수준이 개인의 합병증 검사 수진 행위에 유의한 영향을 미치진 못하였지만 단변량 분석결과에서는 지역의 사회경제적 수준 정도가 중위수 이상일 때 미만일 경우보다 합병증 검사 수진율이 높은 것을 살펴볼 수 있었다.

다수준분석 결과인 <Table 4-5>에 따르면, 당뇨병 환자이지만 주관적 건강수준이 좋을수록, 본인의 혈당이 잘 조절된다고 인지하고 있을수록 건강에 대한 효능감이 높아 합병증에 대한 검사도 많이 할 것이라 기대하였지만 주관적 건강수준이 나쁜 경우와 본인의 혈당이 잘 조절되지 않는다고 인지하는 경우에 합병증 검사수진율이 유의하게 높았다. 또한 현재 진단받은 만성질환의 개수가 많을수록 합병증 검사 수진율이 높았는데 이는 본인의 현재 상태가 좋지 않다고 생각할 때 더 많은 검사를 하는 것으로 유추할 수 있어 환자들에게 질병에 대한 경각심을 가질 수 있도록 하는 접근이 필요하다고 할 수 있다.

혈당 측정횟수가 많아질수록, 본인의 혈당수치를 알고 있을 때, 현재 당뇨병 치료를 받고, 관리교육을 받은 적이 있을 때 합병증 검사 수진율이 높으므로 환자들에게 본인의 질병상태에 관심을 가질 수 있도록 당뇨병에 대한 정보와 관리방법에 대한 적절한 교육이 필요하다고 할 수 있다.

성별에 따라서는 특히, 남성의 경우 흡연자에 있어서 당뇨병 합병증 검사수진율이 유의하게 낮았다. 또한 여성의 합병증 검사 수진행위는 남성에 비해 연령과 소득수준에도 영향을 받았다.

지역수준의 변수들 중에서는 남성과 여성 모두 내과전문의 수가 많은 지역에 거주하고 있을 때 합병증 검사 수진 행위를 할 확률이 1.3 배 높았다. 또한 남성의 경우 도시에 거주할 때 농촌에 거주하는 사람들에 비해 합병증 검사수진 행위를 할 확률이 1.3 배 높았고, 여성의 경우 지역의

대중교통 여건에 대한 만족도가 중위수 이상인 지역에 거주하는 경우 미만인 지역에 비해 합병증 검사 수진 행위를 할 확률이 1.5 배 높았다. 대부분의 의료시설과 의사인력이 도시지역에 집중되어 있어 농촌지역은 도시지역에 비해 보건의료자원에 대한 접근이 어렵다[24]. 의료기관을 이용함에 있어 교통이 불편하고 비용문제로 인해 적절한 치료가 이루어지지 않고 있으며 운동시설등의 문화체육시설이 도시보다 적은 편으로[25], 이로 인해 차이가 발생한다고 할 수 있다. 의료서비스 이용과정에서 교통수단의 발달과 도로시설 정비 등으로 지역간 주민의 이동이 촉진되고 있어 제약요소로 작용하였던 교통으로 인한 문제가 줄어들고 있다고는 하지만[26] 남성은 자가용의존도가 높은 반면, 여성의 경우 남성에 비해 도보나 대중교통을 이용하여 이동하는 경우가 많아[27] 여전히 여성은 대중교통여건에 의해 의료서비스 이용에 영향을 받고 있는 것을 확인할 수 있었다.

이처럼 개인의 당뇨병 합병증 검사 수진행위에 영향을 미치는 요인이 성별에 따라 다르긴 하지만 남성과 여성 모두 본인의 건강상태가 좋지 않다고 인지하고나 질병관리에 적극적인 행동을 취하는 경우, 그리고 지역변수로는 내과전문의 수에 영향을 받았다.

이는 미국의 만성질환 관리모형(Chronic Care Model: CCM)[28]과 WHO 의 혁신적 만성질환 관리전략(Innovative Care for Chronic Conditions Framework)[29]을 벤치마킹한 지역사회와 1 차 의료기관 진료를 기반으로 하는 관리체계[17]로의 접근이 중요하다고 할 수 있다.

만성질환 관리모형(Chronic Care Model:CCM)의 효과에 대한 근거는 여러 연구들에서 제시하고 있다. 국외 연구결과에 따르면 당뇨병환자들을 대상으로 CCM 을 적용한 결과 HbA1c, lipid profile, 요검사, 눈검사, 발검사에서 유의하게 향상된 결과를 보였고[30], 환자의 자가관리를 지지하는 능력이 향상되었으며, 환자에게 제공되는 교육의 횟수도 많았다[31]. 또한 LDL, HbA1c, 혈압감소를 포함하여 당뇨병 관련 결과들이 유의하게 향상되었고, CCM 이 이러한 대상자들의 만성질환 관리에 효과적인 전달체계라고 보았다[32]. 국내 연구결과에 따르면 국내의 심뇌혈관질환 고위험군 등록관리 사업결과 등록환자의 지속치료율과 외래실인원 분포에서 의원급 의료기관의 비중이 증가하였다[33]. 또한 등록환자의 혈압과 혈당수치를 5 년간 추적관찰한 결과 혈압이 유의하게 감소하였고 혈당관리율이 증가하였다[34]. 보건기관의 만성질환 교육 및 홍보정도에 따라 합병증 검사 수진율에는 차이가 없었는데 이는 환자 대상이 아닌 전체 지역주민 대상으로

하는 교육 및 홍보이긴 하나 이들은 언제나 질병을 가질 수 있는 가능성이 있다. 지역주민의 질병관리능력을 향상시킬 수 있도록 효율적이고 적절한 지역사회의 input 이 필요하다고 할 수 있다.

본 연구는 개인수준과 지역수준의 변수를 모두 고려한 다수준 분석을 통해 성별에 따라 개인의 당뇨병 합병증 검사 수진행위에 영향을 미치는 요인이 약간 다르긴 하지만 남성과 여성 모두 본인의 건강상태가 좋지 않다고 인지하거나 질병관리에 적극적인 행동을 취하는 경우, 그리고 지역변수로는 내과전문의 수에 영향을 받는다는 것을 밝혔다. 이는 지역사회와 1차 의료기관의 협업을 통해 당뇨병 관리와 당뇨병에 대한 충분하고 지속적인 학습기회의 제공이 중요하다고 할 수 있다. 지역사회에서는 지역주민들의 질병관리능력을 향상시킬 수 있도록 1차 의료기관과 함께 적절한 체계를 개발하여 지역사회 건강증진 활동을 강화해야 할 것이다.

본 연구에서 사용한 합병증 검사 수진율 변수는 안질환과 신장질환 합병증여부만을 포함하였고 자가응답으로 인한 bias 가 있을 수 있으며, 질병을 미인지하거나 합병증 검사에 대해 미인지하고 있는 경우가 있어 과소추정 되었을 수 있다. 또한 지역의 물리적 환경이나 사회경제적 환경은 구성원의 삶의 질과 건강에 영향을 미치는데[19], 본 연구에서는 지역의 물리적 환경에 대한 변수를 대신하여 지역 주민이 느끼는 자연환경, 생활환경, 교통시설에 대한 주관적인 만족도 변수를 사용하였다. 기후, 환경오염정도, 상하수도와 도로 등 도시기반 시설 등과 같은 지역의 물리적 환경에 대한 변수는 아직 시군구 단위로 데이터를 구축하기엔 정보가 부족하여 추후 이에 대한 객관적 지표를 구성하여 추가적인 연구가 필요할 것이다.

이해상충보고

본 연구는 질병관리본부 지역사회건강조사(2013) 자료를 활용하여 연구되었으며, 본 연구자는 이 연구 수행과 관련하여 직접 혹은 간접적으로 연구의 목적과 상충하는 이해관계가 없음을 밝힙니다.

참고문헌

[1] Kim EY, So A. The effect of a community-based self-help management program for patient with diabetes mellitus. J Korean Acad Community Health Nurs 2009;20(3): 307-215.

[2] Ministry of health and welfare. Korea health statistics 2013: Korea national health and nutrition examination survey(KNHANES VI-1);2014.

[3] Rohlfing CL, et al. Defining the relationship between plasma glucose and HbA1c. Diabetes care 2005; 25(2): 275-278.

[4] The Diabetes Control and Complications Trial Research Group. Lifetime benefits and costs of intensive therapy as practiced in the diabetes control and complications trial. JAMA 1996; 276: 1409-1415.

[5] UK Prospective Diabetes Study (UKPDS) Group. Intensive blood-glucose control with sulphonylureas or insulin compared with conventional treatment and risk of complications in patients with type 2 diabetes. LANCET 1998;352(9131): 837-853.

[6] Stratton IM, et al. Association of glycaemia with macrovascular and microvascular complications of type 2 diabetes (UKPDS 35): prospective observational study. BMJ 2000; 321(7258): 405-412.

[7] Lim S, Kim DJ, Jeong IK, Son HS, Chung CH, Koh G, et al. A nationwide survey about the current status of glysemic control and complication in diabetic patient 2006. Korean Diabetes J 2009; 33: 48-57.
[8] Korean diabetes association. [cited 2015 Nov 13]. Available from: http://www.diabetes.or.kr/general/class/complications.php?code=complication&number=336&mode=view&idx=2.
[9] Korea National Health Information Portal. [cited 2015 Nov 25]. Available from: http://health.mw.go.kr/HealthInfoArea/HealthInfo/View.do?idx=3000&subIdx=2&searchCate=&searchType=&searchKey=&pageNo=&category=&category_code=&dept=&sortType=date&page=1&searchField=&searchWord=.
[10] Korean Diabetes Association. Diabetes fact sheet in Korea 2013 [Internet]. Seoul: Korean Diabetes Association; c2011 [cited 2015 Dec 10]. Available from: http://www.diabetes.or.kr/temp/diabetes_factsheet_2013111.pdf.
[11] Ministry of health and welfare. Community health survey at a glance 2008-2013; 2014.
[12] Kawachi I, Kennedy B, Glass R. Social capital and self-rated health: a contextual analysis. Am J of public health 1999;89(8): 1187-1193.
[13] Kim H, Lee M, Kim H, Lee K, Chang S, Kim V, et al. Factors affecting diabetic screening behavior of Korean adults: a multilevel analysis. Asian Nursing Research 2013; 7: 67-73.
[14] Lee HY, Noh SC. High class statistic analysis-theory and practice. In: Hierarchial linear model. 2nd ed. Goyang: Moonwoo Co.; 2013, p. 423-509.
[15] Kim KH, Chun JH, Sohn HS. The influence of regional deprivation index on personal happiness using multilevel analysis. Epidemiol Health 2015;37:e2015019.
[16] World Health Organization. Global action plan for the prevention and control of non-communicable disease 2013-2020.
[17] Gyeonggi center for hypertension and diabetes. 2013 Gyeonggi center for hypertension and diabetes project report. 2014.

[18] Kim YT, Choi BY, Lee KO, Kim H, Chun JH, Kim SY, et al. Overview of Korean Community Health Survey. Journal of Korean Medical Association 2012; 55(1): 74-83.

[19] Korea Center for Disease and Prevention. Analysis of small area variation of health behavior using 2008 community health survey in Korea. Hallym University & Korea Center for Disease and Prevention, 2010; 6-107.

[20] Seok HS, Kang SH. A study on the regional variation of hypertension medication rate. The journal of digital policy&management 2013; 11(9): 255-265.

[21] Korea center for disease control&prevention. 2008-2013 Local Health statistics at a glance. 2014.

[22] Lee JH, Heo TY. A study of effect on the smoking status using multilevel logistic model. The Korean J of Applied Statistics 2014; 27(1): 89-102.

[23] Kim SH, Lee HJ. Factors affection on outcome indicators in patients with stroke. Health policy and management 2015; 25(1): 31-39.

[24] Lee YJ. Regional distribution and characteristics of health care resources. Journal of the KRSA 2005;21(3): 3-27.

[25] Lee HR. A survey on knowledge of diabetes and self-care behavior of rural patients with diabetes mellitus. Journal of Korean academy of rural health nursing 2013; 8(1): 13-24.

[26] Park SY, Jo EK, Lee KS. How were the regional health care resources used efficiently in Korea?. Korean public health research 2014; 40(4): 107-119.

[27] Kwon KH, Jun MJ. An analysis on the gender differences of life time use and travel mode of Seoul's dual worker households using time use survey data. Journal of the KRSA 2014;30(3):109-129.

[28] Epping-Jordan, et al. Improving the quality of health care for chronic conditions. Qual Saf health Care 2004; 13: 299-305.

[29] WHO. Innovative Care for Chronic Conditions: Building blocks for action. Global report; 2002.

[30] Siminerio LM, Piatt G, Zqibor JC. Implementing the chronic care model for improvements in

diabetes care and education in rural primary care practice. Diabetes Educ 2005; 31(2): 225-234.
[31] Glasgow RE, Funnell MM, Bonomi AE, Davis C, Beckham, V, Wagner EH. Self-management aspects of the improving chronic illness care breakthrough series: implementation with diabetes and heart failure teams. Ann Behav Med 2002; 24(2): 80-87.
[32] Strobel RJ, Gloor B, Freytag S, Riegert-Johnson D, Smith SA, Huschka T, Naessens J, Koeeke TE. Adapting the chronic care model to treat chronic illness at a free medical clinic. J Health Care Poor Underserved 2005; 16(2): 286-296.
[33] Lee HJ, Lee JJ, Hwang TY, Kam s. Development and evaluation of a community staged education program for the cardiocerebrovascular disease high risk patients. J Agric Med Community Health 2012; 37(3): 167-180.
[34] Gyeonggi center for hypertension and diabetes. 2014 Gyeonggi center for hypertension and diabetes project report, 2015.
